# Homogeneity and Viscoelastic Behaviour of Bitumen Film in Asphalt Mixtures Containing RAP

**DOI:** 10.3390/ma14164355

**Published:** 2021-08-04

**Authors:** Adam Liphardt, Piotr Radziszewski, Jan Król

**Affiliations:** Road Construction Technology Department, Faculty of Civil Engineering, Warsaw University of Technology, 00-637 Warsaw, Poland; p.radziszewski@il.pw.edu.pl (P.R.); j.krol@il.pw.edu.pl (J.K.)

**Keywords:** reclaimed asphalt pavement (RAP), staged extraction, dynamic shear rheometer, blending, polymer-modified binders, multiple stress creep recovery (MSCR)

## Abstract

This article discusses the phenomenon of fresh and RAP binders miscibility and presents test results of bitumen film properties from specially prepared asphalt mixtures. The miscibility of a fresh binder and a RAP binder still has not been fully recognised. The aim of this study was to determine the homogeneity level of the bitumen film based on viscoelastic assessment. In addition, an attempt was made to assess the impact of fresh binder on the binders blending degree. The study included assessment of homogeneity of bitumen film comprising various types of bituminous binders. The assessment was conducted on the basis of tests in the dynamic shear rheometer regarding rheological properties of the binders recovered from specific layers of the bitumen film using a staged extraction method. A complex shear modulus as a function of temperature, an elastic recovery R and a non-recoverable creep compliance modulus J_NR_ from MSCR test were determined. The conducted statistical analyses confirmed the significant impact of the type of fresh binder on the blending degree. Regressive dependencies have been set between the differences of the complex shear modulus of the binders subject to mixing and differences of the complex shear modulus of binders from the internal and external layer of the bitumen film comprised of those binders. It was found that there is no full blending of fresh hard bitumen-simulated binder from RAP, which results in non-homogeneity of the bitumen film.

## 1. Introduction

The part of the binder which is involved in the formation of the bitumen film increases with increasing the RAP content in the asphalt mixture. The need to use increasing amounts of RAP forces the necessity to recognise miscibility of binders and outcome properties of binder film in asphalt mixture containing RAP, as well as their impact on asphalt mixture properties.

Current studies indicate that there is no full blending of the RAP binder with virgin binder [1,2,3,4,5,6]. The studies on binder miscibility consider three possible blending scenarios:

I. Full blending—when the binder film is characterised by the same viscoelastic and chemical properties along its thickness;
II. Partial blending—when the properties of the binder are different depending on the point in the binder film, but there is no strict interface between both binders;
III. No blending—when virgin binder covers the RAP binder without blending and the RAP is then referred to as black aggregate or black rock.

**Figure 1:**
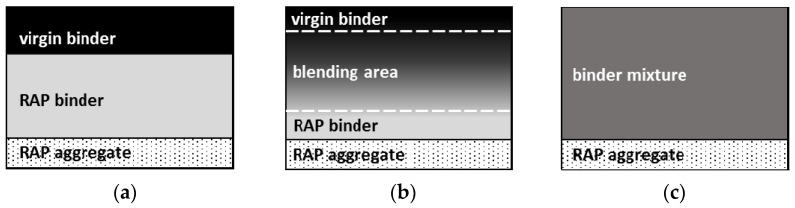
Distribution of virgin binder and RAP binder to the thickness of the binder film depending on the blending degree: (**a**) no blending, (**b**) partial blending, (**c**) full blending [7].

The distribution of binders in the binder film for specific blending degrees is presented in the Figure 1.

The problem of blending between virgin and RAP binders began to be analyzed by researchers in the late 1970s. It was the beginning of intensive development of asphalt recycling technologies [8]. The first works from that period [9,10] nowadays constitute a valuable source of knowledge. Testing methods developed and applied at that time still allow testing of the binder blending phenomenon, but now using much more modern laboratory equipment.

The methods of binder testing miscibility can be divided into two groups. Methods called direct methods constitute the first group. They consist of the assessment of the chemical properties of mixtures or the analysis of the microstructure of the bitumen film. Direct methods include, among others, FTIR spectrographic, GPC gel chromatography, X-ray spectroscopy or microscopy analysis [11,12,13,14,15]. The second group consists of indirect methods. They consist of analysing the viscoelastic parameters of the binder extracted from the bitumen film [16]. Parameters of the binder extracted from the bitumen film are compared with the parameters of the mixtures produced with fully blended binders [17,18]. The method allowing us to obtain binder samples to assess miscibility and homogeneity of the bitumen film is the staged extraction method proposed for the first time by Carpenter and Woloshick in work published in 1980 [9]. That method, based on the staged extraction of the bitumen film layers, leads to separation of samples from the dissolved binder for each of the sub-layers [19,20].

The studies concerning binder blending degree using indirect methods were also conducted by Cooley and Williams with the use of a DSR rheometer to determine the critical temperature with stress of 2.2 kPa. The current analyses of the blending phenomenon prove that in most cases, the most real variant seems to be the indirect variant including a partial blending of both binders [21,22,23,24].

With the discussion of the binders blending phenomenon, the latest scientific research indicates that the activation of the binder in RAP is also a significant and not fully explained problem. The activation of the RAP binder is necessary for its mixing and blending with the virgin binder and virgin aggregate. The degree of binder activation also determines the homogeneity of the asphalt mixture with RAP [25]. Currently, there are no reliable testing methods to predict the RAP binder activation degree. The degree of binder activation and the degree of binder blending are crucial in the design process of asphalt mixtures with RAP. These parameters could be helpful to determine the required content of virgin binder or rejuvenators as well [4]. Previous research shows that the degree of the binder activation and the binder miscibility depends mainly on the mixing temperature while the mixing time is less important [26,27].

This article presents the results of step-by-step research and analyses of the viscoelastic properties of binders extracted from bitumen film from a laboratory-prepared asphalt mixture. The aim of this study was to determine the non-homogeneity level of the bitumen film based on viscoelastic evaluation. In addition, an attempt was made to assess the impact of fresh binder on the blending degree. The assessment was conducted on the basis of tests in a dynamic shear rheometer (DSR) regarding rheological properties of the binders recovered from specific layers of the binder film using staged extraction method.

## 2. Materials and Methods

### 2.1. Materials

Melaphyr aggregate with 2/5 granulation was used to produce the asphalt mixture for the staged extraction procedure. Bitumen film on aggregate was introduced in two layers and was composed of two types of binders—paving bitumen 20/30 simulating RAP binder and virgin binder according to the testing plan. In total, seven types of virgin binders were used, including three paving bitumens, three polymer-modified bitumens (PMB) and one high-modified bitumen (HIMA). In order to ensure the same mixing conditions for all the binders, the temperatures representing dynamic viscosity at the level of 2 Pa·s were specified. The basic properties of the binders used for the tests are presented in Table 1.

### 2.2. Staged Extraction Method

In order to separate two layers of the binder film, the staged extraction method was used. This method was proposed for the first time by Carpenter and Wolosick [6]. That method was based on the staged extraction of the bitumen film layers leading to separation of samples from the dissolved binder for each of the sub-layers. It should be noted that this method does not allow step by step removal of the binder film in layers with the same thickness. However, it can be assumed that the samples obtained in this method from two or three sub-layers allow for an approximate analysis of this phenomenon [28].

In the case of a typical asphalt mixture consisting of aggregates with gradation from 0 to X mm, the fine aggregate particles could clump together in the form of clusters. As a result, a certain amount of binder remains hidden and can be stripped only in complex and time-consuming extraction processes. Considering these limitations and wanting to access the entire specific surface area of the binder as quickly as possible, the one single fraction of aggregate with granulation from 2 to 5 mm was used. In addition, the aggregate used in the mixture was previously washed in a 2 mm sieve in order to eliminate dust and undersized grains. Coarse crushed aggregate with the lowest possible granulation was used in order to introduce as much binder to the mixture as possible while keeping the standard thickness of the binder film.

Asphalt mixture was produced in two steps. In the first step, hot aggregate was covered by 20/30 binder with half of the expected total layer thickness. That way, a system simulating RAP particles was obtained. Next, particles were heated to 140 ± 5 °C corresponding to RAP dosage temperature in hot recycling technology [29]. In the next step, the virgin binder was added, which was followed by the second mixing step lasting for 40 s. Virgin binder used in the second mixing step was heated to the temperature determined on the basis of the viscosity test (2 Pa·s criterium). After mixing, aggregate particles covered by bitumens were scattered on steel trays to cool and provide grains in a loose state.

**Figure 2:**
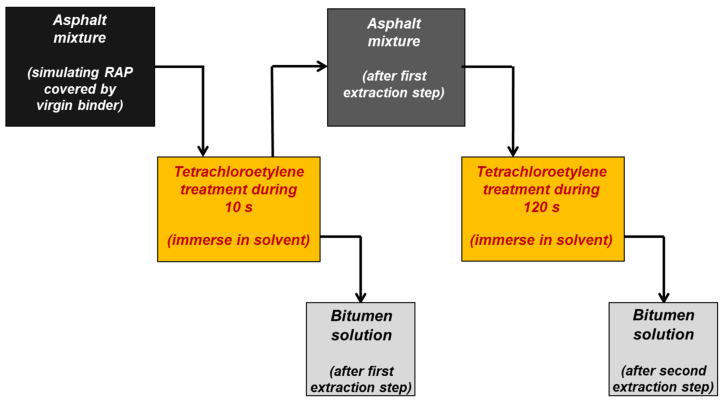
Scheme of the step extraction method.

It was assumed that the amounts of binders obtained from specific layers of the bitumen film should be similar. On the basis of the initial studies, assuming different times needed for extraction of each specific layer, extraction was divided into two stages allowing selection of two sublayers from the whole bitumen film. The first extraction stage of the external layer of the bitumen film lasted for 10 s. The second stage, which aimed to extract the remaining binder, lasted for 120 s. The first extraction stage caused removal of approximately half of the thickness of the bitumen film, while the second stage caused stripping of the remaining part of the binder. The scheme presenting the applied method is included in Figure 2.

The collected bitumen and solvent solutions were evaporated in a vacuum rotary evaporator to recover the pure binder. The recovery process was conducted in accordance with the procedure described in the PN-EN 12697-3 standard. The use of hard binder 20/30 forced conducting the second recovery stage in higher temperature (180 °C) in order to fully vaporise the solvent.

### 2.3. Rheological Tests

Samples of recovered binders were subjected to rheological tests in the Dynamic Shear Rheometer (DSR). Rheometer Physica/Anton Paar MCR 101 (made in Graz in Austria) with Peltier Thermostated Temperature Device P-PTD200 temperature control system was used. The complex shear modulus G* was determined according to the methodology described in the PN-EN 14770 standard. The tests were conducted in two temperature ranges from −5 °C to 25 °C with 10 °C intervals using 8 mm diameter parallel plates with 2 mm gap, and from 30 °C to 100 °C with the same temperature interval using 25 mm parallel diameter plates with 1 mm gap. In both temperature ranges, the tests were conducted with constant angular frequency of 10 rad/s (1.59 Hz). The tests were made in oscillation-controlled strain mode in the range from 0.1% to 15% depending on the test temperature so that the binder remains in a linear viscoelastic range (LVE). For mixtures containing polymer-modified binders, a Multiple Stress Creep Recovery test (MSCR) in temperature of 60 °C was also performed. For the MSCR test, 25 mm diameter parallel plates and 1 mm gap were applied. Tests were conducted for two stress levels, 0.1 kPa and 3.2 kPa, respectively [30].

## 3. Results and Discussion

### 3.1. Complex Modulus and Phase Angle

The degree of blending of the binders was assessed based on an analysis of a difference between the properties of the binder layers extracted and recovered from bitumen film-covered aggregates. In the first step, the zero-miscibility index ΔG*_O_ was determined for original binders (O), i.e., binders before mixing and creation layered bitumen system on aggregate. This index is calculated as a difference of the logarithm of the complex modulus G* of the bitumen simulating the RAP binder (20/30) and logarithm of a complex modulus G* for a bitumen-simulating virgin binder i.e., 35/50, 50/70, 70/100, 10/40–65, 25/55–60, 45/80–55, 45/80–80 (formula—1). The ΔG*_O_ parameter characterizes the maximum possible difference that can occur in the case of complete lack of blending between the two bitumen layers.

(1) $$\Delta G_{O}^{*}=\left|\mathrm{Log}10G_{20/30}^{*}-\mathrm{Log}10G_{\mathrm{lo}}^{*}\right|\;$$

where:

ΔG*_O_—absolute value of the difference of logarithm of a complex modulus of the 20/30 binder and logarithm of a complex modulus of an original virgin binder at a given temperature;

G*_lo_—value of complex modulus of the original virgin binder (35/50, 50/70, 70/100, 10/40–65, 25/55–60, 45/80–55, 45/80–80) at a given temperature [kPa];

G*_20/30_—value of complex modulus of the binder 20/30 at a given temperature [kPa].

The calculated values of ΔG*_O_ index for paving bitumens and for polymer-modified bitumens are presented in Table 2 and Table 3, respectively.

In the second step of the research, a blending assessment was carried out on the specimens with two layers. The binders were recovered by a staged extraction method as was described in Section 2.2. The results of the complex modulus G* for binders from different layers, step by step extracted from laboratory made specimens, are presented in Table 4 and Table 5. The results were organised in the Table 4 and Table 5 into two groups, for the paving bitumens and for the polymer-modified bitumens, respectively. The virgin binder means the binder used to create outer layer of bitumen film. The layer C provided in Table 4 and Table 5 denote layers where complete blending occurred. Results for data series C were obtained by testing physically mixed two binders i.e., 20/30 with other virgin binders, in equal mass proportion.

In order to evaluate the differences between the two bitumen film layers, the ΔG*_R_ index was defined. ΔG*_R_ is the difference between the logarithm of a complex modulus G* of layer 1 (outer) and the logarithm of a complex modulus of layer 2 (inner) at a given temperature. When the binders are fully mixed, the ΔG*_R_ index will have a value of 0.

(2) $$\Delta G^{*}{}_{R}=\left|\mathrm{Log}10G_{w2}^{*}-\mathrm{Log}10G_{w1}^{*}\right|\;$$

where:

ΔG*_R_—absolute value of the difference of logarithm of a complex modulus of the binder of layer 1 and logarithm of a complex modulus of the binder of layer 2 at a given temperature;

G*_w1_—value of complex modulus of a binder of layer 1 (outer) at a given temperature [kPa];

G*_w2_—value of complex modulus of a binder of layer 2 (inter) at a given temperature [kPa].

**Figure 3:**
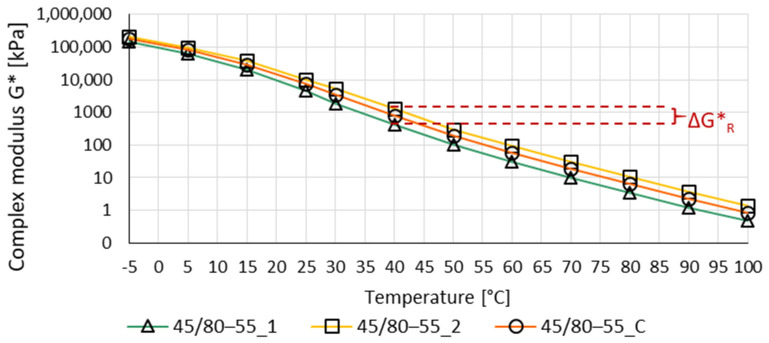
Complex modulus G* for PMB 45/80–55 virgin binder; an example of determining the ΔG*_R_ index. (45/80–55_1—outer layer of bitumen film with virgin binder 45/80–55; 45/80–55_2—inter layer of bitumen film with virgin binder 45/80–55; 45/80–55_C—whole bitumen film with virgin binder 45/80–55).

**Figure 4:**
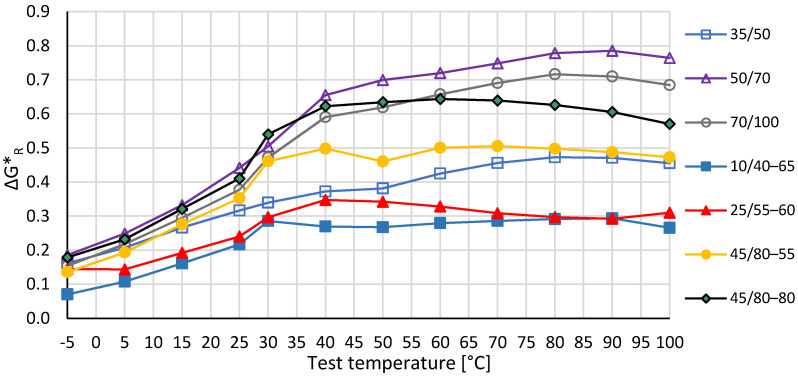
ΔG*_R_ index as a function of temperature, data named in accordance with the fresh binder used as outer layer.

Example dependencies of the complex modulus G* with an example of determination of the ΔG* index are shown in Figure 3. The comparison of the calculated ΔG*_R_ index for all tested recovered binders is shown in Figure 4.

It should be noted that the highest values of ΔG*_R_ index are achieved for paving binders 50/70 and 70/100, and therefore in the case of these binders there are the greatest differences in the complex modulus over the thickness of the bitumen film. The lowest differences of complex shear modulus on the thickness of the bitumen film occur in the case of polymer-modified bitumens i.e., 10/40–65 and 25/55–60. The values of ΔG*_R_ for the remaining analyzed binders have values between those. On the basis of the obtained results, it can be concluded that the difference between the complex modulus of both layers of the bitumen film decreases with an increase of the binder stiffness and presence of the polymer. In addition, it was stated that the value of ΔG*_R_ for all analysed binders increases with an increase of the test temperature in the range from −5 to approximately 40–50 °C. In higher temperatures (50–100 °C), such an increase was observed only in the case of paving bitumen, in particular for 50/70 and 70/100. The value of the indexes for modified binders in the temperature range 40–100 °C are almost constant at a similar level. In addition, it was stated that the value of ΔG*_R_ does not have a linear relationship to the layer differences, so that the gap between curves in Figure 4 at different temperatures shows different layer differences. In order to determine the statistical significance of the impact of the type of the binders, polymer modification and test temperature on the ΔG*_R_ index, an ANOVA analysis was conducted, with the level of probalility 95% [14]. When p values are less than or equal to 0.05 value it could be interpreted that there is a significant impact of the independent variable on the tested value. The results of the ANOVA are presented in Table 6. The conducted ANOVA analysis proved that the value of ΔG*_R_ significantly depends on the test temperature and the type of virgin binder used in the layered aggregate/bitumen system.

It was found that in the case of paving bitumens, the values of the ΔG*_O_ index are quite similar to the values obtained for the binders from the step extraction method. For the original polymer-modified bitumen, the values of ΔG*_O_ indicate higher variability in the function of the temperature, but the obtained ΔG*_O_ values are comparable with the ΔG*_R_ values calculated for binders extracted from layers.

Therefore, it can be concluded that in the case when a full blending between two bitumen layers does not exist, the differences of G* of the internal and external binder film layer will approximately correspond to the differences of G* for both binders being mixed. In order to verify those findings, an analysis of correlation was conducted for original binders and recovered binders. The analysis used the following assessment criteria:

r < 0.5 low correlation

0.5 ≤ r < 0.7 average correlation

0.7 ≤ r < 0.9 high correlation

0.9 ≤ r < 1 almost full correlation.

The results of the correlation analysis of ΔG* indexes are presented in Table 7. Addtionally, results are arranged separately in the group of paving bitumens and polymer-modified bitumen, and jointly in the group of all binders. The correlation analysis did not include high-modified binder because of a different nature in the change of the complex modulus in the function of the temperature in relation to other polymer-modified binders.

The results of the analysis showed that the differences in the value of the complex modulus in the range from −5 °C to +100 °C for external and internal layers significantly correlate with the differences of the complex modulus for the original binders tested in the same temperature range (*p* = 0.000). In the case of paving bitumens, almost full correlation was found with the r Pearson value equal to 0.948. In the group of all analysed virgin binders, correlation was determined on a high level. It can be assumed that the low correlation in the case of polymer-modified bitumen may be related to the differences in the content and cross-linking degree of polymer in the tested extracted binders. Moreover, the low correlation in the case of polymer-modified bitumen may be related to the higher viscosity of polymer-modified bitumen. As a result, the thickness of the film with polymer-modified bitumen can be more varied. The consequence is a wider dispersion of the results. 

**Figure 5:**
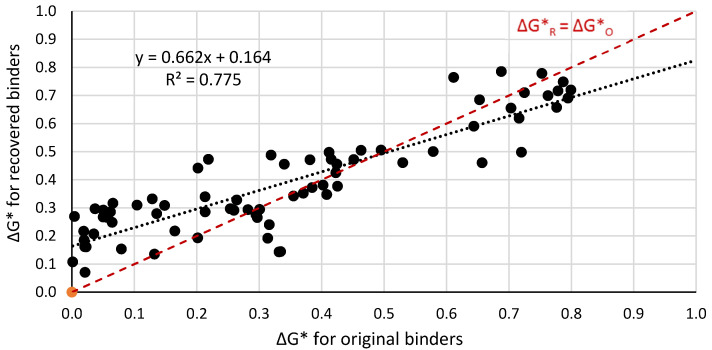
Correlation of the ΔG*_R_ index for extracted binders and ΔG*_O_ index for original binders in the temperature range from −5 °C to +100 °C in the group of all bitumens.

**Figure 6:**
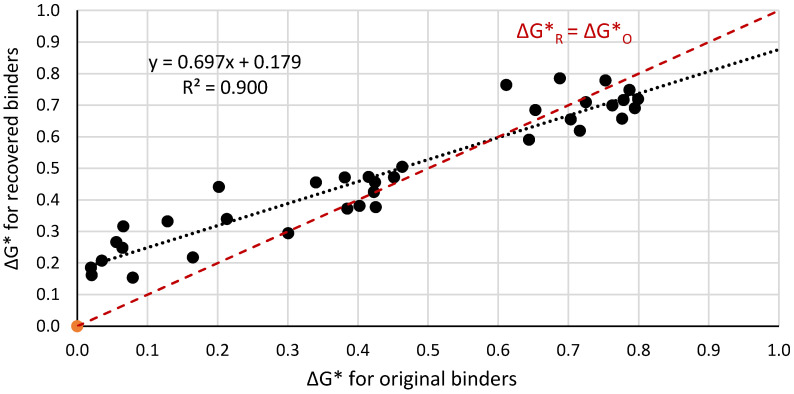
Correlation of the ΔG*_R_ index for extracted binders and ΔG*_O_ index for original binders in the temperature range from −5 °C to +100 °C limited only to the group of paving bitumens.

**Figure 7:**
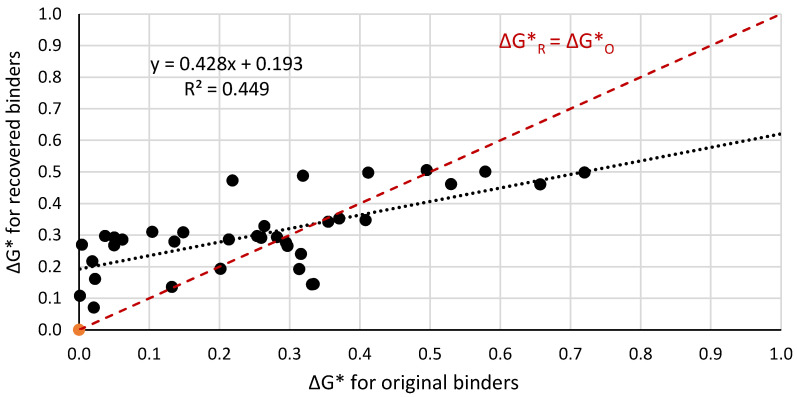
Correlation of the ΔG*_R_ index for extracted binders and ΔG*_O_ index for original binders in the temperature range from −5 °C to +100 °C limited only to the group of polymer-modified bitumens.

Figure 5, Figure 6 and Figure 7 present a graphical interpretation of the correlation analysis for ΔG* indexes.

The statistical analysis of the correlation force demonstrated that increase of the differences of the complex modulus G* of the mixed binders causes an increase in the difference between the complex modulus of binders extracted from two selected layers of the bitumen film. In Figure 5, Figure 6 and Figure 7, the red line represents the case when ΔG*_R_ could be equal to ΔG*_O_, i.e., extracted binders characterised in non miscibility. On the basis of the position of the regression line in relation to the red one, it allows us to conclude that with the increase of the value of ΔG*, the differences between the values of these indexes for the extracted and original binders increase. The smallest differences are observed for paving binders. Based on the statistical analysis, the dependence between the value of the ΔG*_O_ index (original binders) and the value of the ΔG*_R_ index (recovered binder) was determined. The dependence including results for all analysed binders is presented in Equation (3).

ΔG*_R_ = 0.662ΔG*_O_ + 0.164 (3)

On the basis of the Equation (3), it can be stated that the differences between logarithms of the complex modulus of an internal and external layer of the bitumen film constitute approximately 66% of the difference of the complex modulus for original binders used in binder film. The value of the slope factor of line function lower than 1 should be interpreted as the impact of partial blending of both binders. As a result of partial blending, the properties of the external layer change in the direction of the properties of the internal layer, and simultaneously the properties of the internal layer change in the direction of the properties of the external layer. In the case of the complete lack of blending of the two binders, the slope factor value will equal 1. The constant in the equation of about 0.16 kPa is related to the stifeness aging effect. The binder, which simulates rap asphalt, has stiffened due to aging. Aging took place when the 20/30 binder was mixed with aggregate and when the RAP-simulating mixture was reheated during mixing with fresh binder. Consequently, the greater stiffness of the inner layer causes a greater difference in the case of recovered binders. In a specific case when both original binders creating the bitumen film are characterised by the same values of complex shear modulus (ΔG*o = 0), the determined value of ΔG* will be approximately 0.16 kPa. This could be expected in real conditions, if the properties of the binder recovered from RAP and the original virgin binder were analyzed. Then, the constant could be equal to 0.

Equation (4) describes a similar relationship for virgin binders from the group of paving bitumens, while the group of polymer-modified bitumens is described by Equation (5).

ΔG*_R_ = 0.697ΔG*_O_ + 0.179 (4)

ΔG*_R_= 0.428ΔG*_O_ + 0.193 (5)

Based on comparison of the values of slope factors for paving bitumen (0.697) and polymer-modified bitumen (0.428), it can be stated that binder film composed of two paving bitumens is characterized by a lower level of homogeneity than binder film consisting of one of the layers made by polymer-modified bitumens. The specified dependencies can be applied in practice to forecast a non-homogeneity in the bitumen film composed of two types of binders with a different complexed modulus. Especially, this could be applied when reclaimed asphalt pavement is used.

### 3.2. Multiple Stress Creep Recovery Test—MSCR

**Figure 8:**
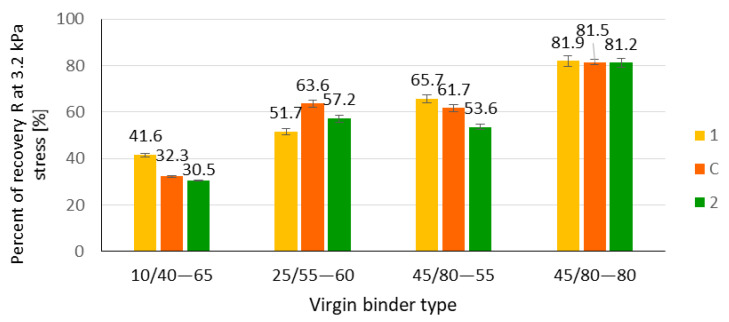
Percent recovery R at 3.2 kPa stress; 1—outer layer of binder film, 2—inner layer of binder film, C—complete blending.

**Figure 9:**
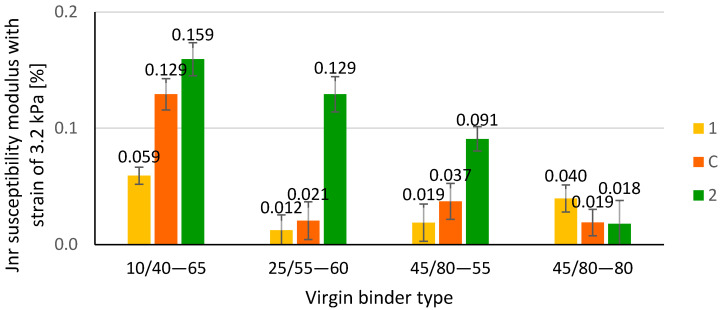
Non-recoverable creep compliance J_NR_ at 3.2 kPa stress; 1—outer layer of binder film, 2—inner layer of binder film, C—complete blending.

In a further stage of the studies, extracted binders from bitumen film containing PMBs were subjected to the multiple stress creep recovery tests (MSCR) which were introduced to characterise how elastic properties of PMBs change in the thickness of the bitumen film. A comparison of tested values of R3.2 and JNR3.2 is presented, as appropriate, in Figure 8 and Figure 9.

In order to determine the significance of differences in parameters R_3,2_ and J_NR3,2_ for specific layers of the bitumen film, as well as types of applied fresh binders, an ANOVA analysis was conducted. The results of variance analysis are presented in Table 8.

While analysing the values of parameter R_3,2_ presented in Figure 8 and results of variance analysis presented in Table 8, it can be concluded that in the case of regular polymer-modified bitumen, there are significant differences between the values calculated for external, internal and entire layers of the bitumen film. These differences indicate the non-homogenity of the bitumen film in terms of its elastic properties. It can be concluded that both binders are not fully blended. The external layer of the bitumen film, which contains mainly polymer-modified bitumen, is characterised by a higher elastic recovery. It should be noted that in the case of the tested binders recovered from a mixture containing high-polymer-modified bitumen 45/80–80, there were no found significant differences between internal and external layers of the bitumen film. It can be assumed that the elastic properties of the internal layer were changed by the modifier. It should be noted that HIMA binder is characterised by a softer bitumen base and a higher amount of polymer used for production than in traditional polymer-modified bitumen. In addition, some changes in elastic properties were recognised for internal layers of bitumen film, depending on the type of used binder.

While analysing the values of creep compliance (J_NR3,2_) presented in Figure 9 and the ANOVA results, it can also be stated that in the case of regular polymer-modified bitumens, there are significant differences between the values calculated for external and internal layers of the bitumen film. The lowest values of creep compliance occured for the external layer of the binder film (virgin polymer-modified binder) and the lowest for the external layer (paving bitumen). Similarly, as in the case of elastic recovery (R_3,2_ parameter), the found differences proved non-homogenity of the bitumen film in terms of its creep properties.

## 4. Conclusions

Summarising the conducted analyses within the scope of assessment of homogeneity of bitumen film in asphalt mixture systems, it should be stated that as a result of lack of full blending of two bituminous binders, the bitumen film covering the aggregates is not homogenous. These findings result from variability of viscoelastic properties along the thickness of bitumen film. On the basis of the conducted laboratory tests and analyses, the following detailed conclusions can be formulated:

- The internal layer of the bitumen film has properties similar to the binder-simulated RAP cluster, while the external layer has properties similar to fresh binder. The presented non-homogeneity of bitumen film proves no full blending of the binders is included in it.
- The ΔG* index allows for assessment of homogeneity of bitumen film based on the G* complex modulus from DSR tests. The binders from different bitumen layers should be extracted step by step. Thus, this indirectly allows us to assess the degree of blending of fresh binder and binder from RAP.
- In medium and high operating temperatures, the differences of viscoelastic properties along the thickness of the bitumen film increase together with an increase of the differences of properties of binders subjected to mixing. Statistical regression was set between the differences of complex modulus of the binders subjected to mixing and differences of complex modulus of binders come from the internal and the external layers of the bitumen film layers comprised of those binders.
- The MSCR test analysis allowed us to recognise that the bitumen film in an asphalt mixture system varies in terms of elastic recovery and creep compliance properties, when polymer-modified bitumens are used.
- The staged extraction method proposed in this work can also be used to detect the presence of RAP in asphalt mixture. Currently, RAP in asphalt mixtures is mainly detected on the basis of petrographic analysis of the mineral aggregates. However, such analysis could be ineffective if RAP and virgin aggregates were characterised by the same petrographic stock. In such a case, binder properties analysis is the only option to detect RAP. Because of the proven non-homogeneity of the bitumen film in an asphalt mixture system, the presented method can be an effective tool to detect whether the asphalt mixture contains RAP.

## Figures and Tables

**Table 1:** Basic properties of the binders.

Binder Type		Penetration (0.1 mm)	Softening Point (R&B) [°C]	Dynamic Viscosity in Temperature: (Pa·s)		
				90 °C	110 °C	135 °C
Paving bitumen	20/30	23	63.0	57.9	8.3	1.5
	35/50	41	55.0	22.1	3.9	0.7
	50/70	62	49.0	10.9	2.1	0.5
	70/100	85	46.0	6.7	1.4	0.3
PMB	10/40–65	33	72.0	106.6	11.9	2.0
	25/55–60	31	69.6	126.6	14.2	2.2
	45/80–55	60	67.6	62.6	7.0	1.2
HIMA	45/80–80	51	89.6	129.5	13.6	1.7

**Table 2:** ΔG*_O_ index for the original and after staged extraction-recovered paving bitumens.

Virgin Binder Type	Binder Film Layer	ΔG*_O_ Index (kPa)											
		Temperature (°C)											
		−5	5	15	25	30	40	50	60	70	80	90	100
35/50	original	0.161	0.207	0.266	0.316	0.339	0.372	0.381	0.425	0.456	0.473	0.471	0.455
	recovery	0.021	0.035	0.056	0.066	0.213	0.385	0.402	0.423	0.424	0.415	0.381	0.340
50/70	original	0.185	0.248	0.332	0.441	0.505	0.655	0.699	0.720	0.748	0.778	0.785	0.764
	recovery	0.019	0.064	0.129	0.202	0.463	0.703	0.763	0.799	0.787	0.753	0.688	0.611
70/100	original	0.154	0.218	0.295	0.377	0.472	0.591	0.619	0.657	0.691	0.716	0.710	0.685
	recovery	0.079	0.165	0.301	0.425	0.451	0.644	0.716	0.776	0.795	0.779	0.725	0.653

**Table 3:** ΔG*_O_ index for the original and after staged extraction recovered polymer-modified bitumens.

Virgin Binder Type	Binder Film Layer	ΔG*_O_ Index (kPa)											
		Temperature (°C)											
		−5	5	15	25	30	40	50	60	70	80	90	100
10/40–65	original	0.070	0.108	0.161	0.217	0.286	0.269	0.267	0.279	0.286	0.291	0.293	0.266
	recovery	0.021	0.001	0.023	0.019	0.062	0.004	0.050	0.136	0.213	0.259	0.282	0.297
25/55–60	original	0.145	0.144	0.192	0.240	0.297	0.347	0.342	0.328	0.308	0.297	0.292	0.310
	recovery	0.334	0.332	0.314	0.316	0.254	0.408	0.355	0.264	0.148	0.037	0.050	0.104
45/80–55	original	0.136	0.193	0.276	0.352	0.461	0.498	0.460	0.501	0.505	0.498	0.488	0.473
	recovery	0.132	0.202	0.295	0.371	0.530	0.720	0.657	0.579	0.495	0.412	0.319	0.219
45/80–80	original	0.179	0.232	0.321	0.410	0.690	0.623	0.634	0.644	0.639	0.626	0.605	0.570
	recovery	0.235	0.366	0.549	0.690	0.699	0.765	0.615	0.409	0.157	0.114	0.371	0.584

**Table 4:** Complex shear modulus G* of the paving bitumens as a virgin binder.

Virgin Binder Type	Layer	Complex Modulus G* (kPa)											
		Temperature (°C)											
		−5	5	15	25	30	40	50	60	70	80	90	100
35/50	1	270,000	117,640	42,000	10,580	4328	991	248	71	21	6.2	2.0	0.7
	2	391,600	189,680	77,560	21,920	9455	2335	598	189	59	19	6.0	2.0
	C	296,800	136,200	51,760	14,672	6144	1536	415	132	41	13	4.2	1.5
50/70	1	166,400	762,00	28,420	7514	3410	748	184	54	16	4.8	1.5	0.5
	2	255,000	135,000	61,040	20,760	10,900	3384	922	284	89	29	9.2	3.0
	C	229,000	113,000	46,240	13,800	6562	1734	423	127	40	12	3.9	1.3
70/100	1	167,200	73,300	26,220	6682	2686	584	140	39	11	3.2	1.0	0.4
	2	238,200	121,000	51,660	15,920	7960	2274	584	178	54	17	5.3	1.7
	C	174,000	83,200	32,680	9348	4842	1172	275	84	25	7.6	2.3	0.8

**Table 5:** Complex shear modulus G* of the PMBs and HIMA binders as a virgin binder.

Virgin Binder Type	Layer	Complex Modulus G* [kPa]											
		Temperature [°C]											
		−5	5	15	25	30	40	50	60	70	80	90	100
10/40–65	1	175,000	74,280	24,180	5704	2176	482	115	32	9.8	3.1	1.0	0.4
	2	205,800	95,180	35,040	9398	4200	896	213	62	19	6.0	1.9	0.7
	C	162,400	70,060	23,580	5836	2608	552	134	38	11	3.6	1.2	0.4
25/55–60	1	79,157	41,041	17,000	4355	1942	471	117	34	11	3.7	1.2	0.4
	2	110,458	57,110	26,460	7570	3844	1048	258	73	23	7.3	2.4	0.8
	C	95,749	49,568	20,784	5641	2703	702	174	50	16	5.3	1.8	0.7
45/80–55	1	149,000	62,500	19,900	4612	1902	416	104	30	9.9	3.4	1.2	0.5
	2	203,600	97,500	37,560	10,380	5498	1312	301	97	32	11	3.8	1.4
	C	183,000	81,660	29,080	7458	3558	769	190	58	19	6.4	2.3	0.9
45/80–80	1	125,200	47,780	14,040	2998	871	213	58	20	7.8	3.2	1.4	0.6
	2	189,000	81,480	29,400	7713	4265	894	253	90	34	14	5.7	2.4
	C	154,400	65,300	22,720	5622	2062	485	132	46	17	7.0	2.9	1.2

**Table 6:** Results of the ANOVA for ΔG*_R_ index and type of the binders.

Dependent Variable	Qualitative Factors					
	Virgin Binder Type		Virgin Binder Modification (Paving Binders/PMBs)		Test Temperature (−5–100 °C)	
	*p*	Relevance	*p*	Relevance	*p*	Relevance
ΔG*_R_	0.000	significant	0.001	significant	0.000	significant

**Table 7:** Statistical analysis, the correlation of ΔG* indexes of extracted and original binders.

Parameters	Paving Bitumen	PMB	Paving Bitumen and PMB
r (Pearson)	0.948	0.670	0.881
R^2^	0.900	0.449	0.775
Correlation power	almost full	low	high
*p*	0.000	0.000	0.000
Correlation relevance	relevant	relevant	relevant

**Table 8:** Summary of the ANOVA analysis for the R _3,2_ and J_NR 3,2_ parameters.

Dependent Variable	Qualitative Factors			
	Virgin Binder Type		Layer (Inter, Outer) of Binder Film	
	*p*	Relevance	*p*	Relevance
R_3,2_	0.000	very relevant	0.001	very relevant
J_NR3,2_	0.000	very relevant	0.001	very relevant

## Data Availability

Main Library of Warsaw University of Technology—Ph.D. Thesis (in Polish) “Ocena mieszalności lepiszczy w aspekcie stosowania destruktu asfaltowego do mieszanek mineralno-asfaltowych” Adam Liphardt. https://repo.pw.edu.pl/info/phd/WUT750d5a6010e14b6da59a66e6c27727d2/.
